# Mr. Francis Richard Gibbs

**Published:** 1911-04-22

**Authors:** 


Mr. Francis Richard Gibbs, of Claytons, Bourne End, has died in London, aged forty-two. He studied at St. Mary's Hospital, took his diploma in 1894, and had held the post of resident casualty officer at St. Mary's. His other appointments included those of Medical Officer to the Woolrun District Wycombe Union and to the Foresters. He was also medical referee to several assurance companies.

